# Europium-doped amorphous calcium phosphate porous nanospheres: preparation and application as luminescent drug carriers

**DOI:** 10.1186/1556-276X-6-67

**Published:** 2011-01-12

**Authors:** Feng Chen, Ying-Jie Zhu, Kui-Hua Zhang, Jin Wu, Ke-Wei Wang, Qi-Li Tang, Xiu-Mei Mo

**Affiliations:** 1 State Key Laboratory of High Performance Ceramics and Superfine Microstructure, Shanghai Institute of Ceramics, Chinese Academy of Sciences, Shanghai 200050, P. R. China; 2 College of Chemistry, Chemical Engineering and Biotechnology, Donghua University, Shanghai, 201620, P. R. China

## Abstract

Calcium phosphate is the most important inorganic constituent of biological tissues, and synthetic calcium phosphate has been widely used as biomaterials. In this study, a facile method has been developed for the fabrication of amorphous calcium phosphate (ACP)/polylactide-block-monomethoxy(polyethyleneglycol) hybrid nanoparticles and ACP porous nanospheres. Europium-doping is performed to enable photoluminescence (PL) function of ACP porous nanospheres. A high specific surface area of the europium-doped ACP (Eu^3+^:ACP) porous nanospheres is achieved (126.7 m^2^/g). PL properties of Eu^3+^:ACP porous nanospheres are investigated, and the most intense peak at 612 nm is observed at 5 mol% Eu^3+ ^doping. *In vitro *cytotoxicity experiments indicate that the as-prepared Eu^3+^:ACP porous nanospheres are biocompatible. *In vitro *drug release experiments indicate that the ibuprofen-loaded Eu^3+^:ACP porous nanospheres show a slow and sustained drug release in simulated body fluid. We have found that the cumulative amount of released drug has a linear relationship with the natural logarithm of release time (*ln*(*t*)). The Eu^3+^:ACP porous nanospheres are bioactive, and can transform to hydroxyapatite during drug release. The PL properties of drug-loaded nanocarriers before and after drug release are also investigated.

## Introduction

The development of multifunctional nanosystems, for maximum therapeutic benefit including early diagnoses of the diseases and delivery of suitable therapeutic drugs, holds a promise for the future of clinical treatment to enhance therapeutic efficacy [1,2]. Therefore, it is highly desirable to develop novel methods that can achieve simultaneous *in vivo *imaging and treatment based on nanotechnology. Nanoparticles show unique size-dependant physical and chemical properties and have great potential for clinical use [3,4]. Dual or multifunctional nanosystems can be constructed based on nanostructures with well-designed structures and constituents, which are simultaneously capable of diagnosis and treatment [5]. Many kinds of nanostructures have been fabricated from a multitude of materials and used in bio-imaging, including quantum dots [6-8], silica particles [9], gold [10], carbon nanotubes [11], dendrimers [12-14], magnetic nanoparticles [15-18], and so on. However, in the search for the nanosystems for bio-imaging and therapy, a couple of the major concerns are their biodegradability and toxicity [19-21]. To date, the development of multifunctional inorganic nanosystems with both biocompatible and biodegradable properties has been little reported, and more research is needed in this regard.

Calcium phosphates including hydroxyapatite (HAp) are the most important inorganic constituents of biological tissues such as bone and tooth [22-25]. Thus, synthetic calcium phosphates are of great significance because of their biodegradability and biocompatibility, and have been investigated for applications in bone repair/tissue engineering [26,27], drug loading and release [28,29], gene delivery [30,31], and other biomedical areas. Owing to its chemical nature, calcium phosphate nanostructures may serve as an ideal candidate for both bio-imaging and drug delivery. The investigation on rare earth-doped calcium phosphates has become a hot research topic [32-37]. However, up to now, little work has been reported on dual or multifunctional calcium phosphate nanostructures for biomedical applications. HAp, a kind of chemically stable calcium phosphate, has been used for drug storage and luminescence [38]. The development of bi-functional nanosystems of other calcium phosphates such as amorphous calcium phosphate (ACP) is still scarce. Compared with HAp, ACP is bioactive with better biodegradability, and can promote osteoblast adhesion and osteoconductivity [39,40].

In this article, the authors report a facile method for the fabrication of ACP/polylactide-block-monomethoxy(polyethyleneglycol) (PLA-mPEG) hybrid nanoparticles and ACP porous nanospheres. ACP is an important kind of calcium phosphate and is present in natural bone [41]. PEG and PLA are widely used as biocompatible polymers and currently possess the approval of the US Food and Drug Administration for use in a variety of biomaterials applications [42,43]. Europium doping was performed to enable photoluminescence (PL) function of ACP porous nanospheres. *In vitro *cytotoxicity experiments showed that the as-prepared porous nanospheres were biocompatible. *In vitro *drug loading and release experiments indicated that ibuprofen-loaded europium-doped ACP (Eu^3+^:ACP) porous nanospheres had a slow and sustained drug release profile in simulated body fluid (SBF). The cumulative amount of the released drug had a linear relationship with the natural logarithm of release time (*ln*(*t*)). The Eu^3+^:ACP porous nanospheres were bioactive, and could transform to HAp after drug release.

### Experimental conditions

The block copolymer PLA-mPEG (*M*_w _= 8000 Da) was purchased from Jinan Daigang Co., Ltd., Jinan, China, and the molecular weight of the PEG segment was 5000 Da. Other chemicals were purchased from Sinopharm Chemical Reagent Co., Shanghai, China, and used as received without further purification. For the preparation of ACP/PLA-mPEG hybrid nanoparticles, 1.775 g of Na_2_HPO_4 _12H_2_O and 0.025 g of PLA-mPEG was dissolved in 60 mL of distilled water, and the pH value was adjusted to 10 using ammonia, followed by magnetic stirring for 1 h to form a clear solution. Then, 60 mL of calcium chloride aqueous solution containing 0.33 g of CaCl_2 _and 0.025 g of PLA-mPEG was added dropwise (10 mL/min) to the above solution, and the pH value was maintained at 10 by slow addition of ammonia. The white precipitate was collected and washed by centrifugation-redispersion cycles with distilled water and ethanol. For the preparation of ACP porous nanospheres, 0.1 g of the as-obtained precipitate was transferred into a 70-mL Teflon autoclave with 50 mL *N*, *N*-dimethylformamide (DMF) and treated at 200°C for 1 h by a microwave-solvothermal system (MDS-10, Sineo, Shanghai, China). The product was washed with distilled water and ethanol and dried at 60°C. For the preparation of europium-doped Eu^3+^:ACP porous nanospheres, europium nitrate aqueous solution was added into the calcium source solution before mixing with phosphate source solution. The doping concentration of Eu^3+ ^was 1, 5, and 10 mol% relative to Ca^2+^.

PL measurements of Eu^3+^:ACP porous nanospheres were carried out on a spectrofluorometer (Fluorolog-3, Jobin Yvon, France) at room temperature.

Porcine iliac artery endothelial cells (PIECs) were obtained from the Institute of Biochemistry and Cell Biology (Chinese Academy of Sciences). All the culture media and reagents used in cytotoxicity tests were purchased from Gibco Life Technologies (USA). PIECs were seeded in 96-well flat-bottom microassay plates at a concentration of 2 × 10^4 ^cells/mL and cultured with 200 μL/well Dulbecco's Modified Eagle's Medium (DMEM) with 10% fetal serum, 100 U/mL penicillin, and 100 U/mL streptomycin for 48 h in a humidified incubator (BB-15, Heraeus, Germany) with 5% CO_2 _at 37°C. The samples were diluted with DMEM without fetal bovine serum at a concentration of 6 mg/mL. Then, 50, 100, and 150 μL of each sample solution was added into each well, and the cells were cultured for 24 h. To evaluate cytotoxicity, cell viability was quantified using 3-(4,5-dimethylthiazol-2-yl)-2,5-diphenyltetrazolium bromide assay (Sigma, USA) and Enzyme-labeled Instrument (MK3, Thermo, USA). Data were measured in three independent parallel experiments, and all data points were plotted as means ± standard deviation (SD) (*n *= 3).

The typical drug loading and *in vitro *drug release experiments were performed as follows: 0.5 g of dried powder of Eu^3+^:ACP porous nanospheres was added into 50 mL hexane solution with an ibuprofen concentration of 40 mg/mL. The suspension was then stirred for 24 h in a sealed vessel at 37°C. The Eu^3+^:ACP porous nanospheres with loaded drug was centrifuged, and 2 mL supernatant was analyzed by UV-Vis absorption spectroscopy at a wavelength of 263 nm to calculate the ibuprofen storage. The drug loaded Eu^3+^:ACP porous nanospheres were washed with fresh hexane, dried at 60°C in air, and compacted into disks (0.3 g each, diameter 10 mm) at a pressure of 3 MPa. Each disk was immersed into 200 mL of SBF at 37°C under shaking at a constant rate using a desk-type oscillator (THI-92A, China). Two milliliter of solution was removed for UV-Vis analysis at 263 nm at given time intervals to measure the amount of ibuprofen released, and this quantity of solution was replaced with the same volume of fresh SBF.

Transmission electron microscopy (TEM) micrographs were obtained using a JEOL JEM 2100 field emission electron microscope. X-ray diffraction (XRD) patterns were recorded using a Rigaku D/max 2550 V X-ray diffractometer with a graphite monochromator (Cu K_*α *_radiation, λ = 1.54178 Å). Thermogravimetry (TG) curves were obtained at a heating rate of 10°C in nitrogen using a STA 409/PC simultaneous thermal analyzer (Netzsch, Germany). The Brunauer-Emmett-Teller (BET) surface area and pore size distribution were measured with an accelerated surface area and porosimetry system (ASAP 2010).

## Results and discussion

**Figure 1:**
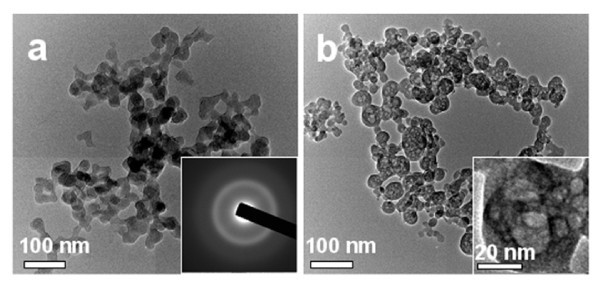
**TEM micrographs**: **(a) **the as-prepared ACP/PLA-mPEG hybrid nanoparticles; and **(b) **ACP porous nanospheres.

**Figure 2:**
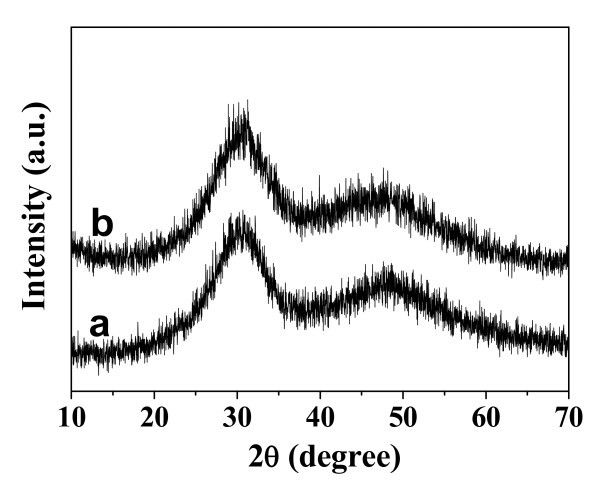
**XRD patterns**:**(a) **the as-prepared ACP/PLA-mPEG hybrid nanoparticles; and **(b) **ACP porous nanospheres.

The morphology of the as-prepared hybrid ACP/PLA-mPEG and ACP were investigated with TEM (Figure 1). From Figure 1a, one can see that the as-prepared hybrid ACP/PLA-mPEG consists of nanoparticles with diameters in the range of 10-60 nm. In some cases, nanoparticles with a porous structure were also observed as a minor product. The selected area electron diffraction pattern of the as-prepared ACP/PLA-mPEG hybrid nanoparticles shows an electron diffraction pattern of amorphous structure (inset of Figure 1a). In contrast, the TEM micrograph of ACP prepared by microwave-solvothermal treatment of ACP/PLA-mPEG hybrid nanoparticles in DMF presents nanospheres with a porous structure (Figure 1b). The magnified TEM image (inset of Figure 1b) of a single ACP nanosphere shows the nanopores in the nanosphere. The sizes of ACP porous nanospheres are similar to those of ACP/PLA-mPEG hybrid nanoparticles, indicating no significant growth of ACP porous nanospheres during microwave-solvothermal treatment of hybrid ACP/PLA-mPEG in DMF. The XRD pattern of the as-prepared ACP/PLA-mPEG hybrid nanoparticles (Figure 2a) shows no discernable peaks of crystalline calcium phosphate but a characteristic hump of amorphous phase at around 2θ = 30°, indicating that the sample consisted of ACP [44]. Similarly, the XRD pattern of ACP porous nanospheres (Figure 2b) indicates that the sample consisted of ACP, too. Energy dispersive spectroscopy (EDS) measurements were also performed to analyze the chemical composition of the sample. The EDS data show that the chemical composition of the sample is essentially uniform in the sample. The molar ratio of Ca to P was measured to be 1.41, and the molar ratio of (Eu + Ca) to P was 1.49. The molar ratio of Eu^3+ ^to Ca^2+ ^was 5.3%, which is close to the nominal composition.

**Figure 3:**
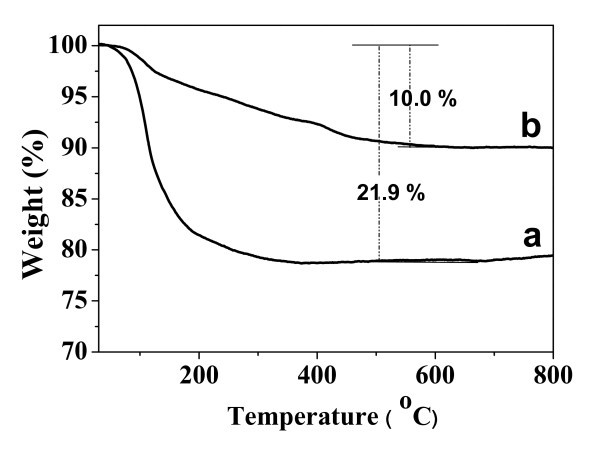
**TG curves**: **(a) **the as-prepared ACP/PLA-mPEG hybrid nanoparticles; and **(b) **ACP porous nanospheres.

In order to determine the content of PLA-mPEG in the ACP/PLA-mPEG hybrid nanoparticles, TG analysis was employed. The TG curve of ACP/PLA-mPEG hybrid nanoparticles is displayed in Figure 3; the weight loss before about 100°C can be assigned to the adsorbed water, while the weight loss between 100 and 425°C is caused by the decomposition of the polymer segments. For the TG curve of ACP porous nanospheres, the weight loss is also caused by adsorbed water and crystal water. On the basis of the analyses of TG curves, the total weight losses of ACP/PLA-mPEG hybrid nanoparticles and ACP porous nanospheres are about 21.9 and 10%, respectively. The weight percentage of PLA-mPEG in the as-prepared ACP/PLA-mPEG hybrid nanoparticles is estimated to be approximately 11.9%.

**Figure 4:**
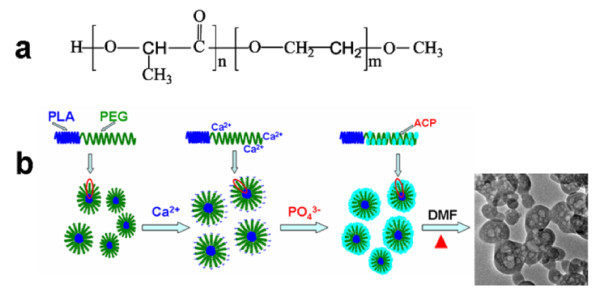
**Illustration of the strategy for the preparation of ACP/PLA-mPEG hybrid nanoparticles and ACP porous nanospheres**: **(a) **Chemical structure of PLA-mPEG and **(b) **the proposed formation mechanism of ACP/PLA-mPEG hybrid nanoparticles and ACP porous nanospheres.

It was reported that the negatively charged oxygen atoms of C-O-C groups in PEG could interact with divalent cations such as Ca^2+ ^via electrostatic attraction [45,46], and hence, the Ca^2+^/PEG complex was formed. The chemical structure of PLA-mPEG is illustrated in Figure 4a; we propose that Ca^2+ ^ions can bind with PEG segments of PLA-mPEG. Therefore, the formation of ACP/PLA-mPEG hybrid nanoparticles may follow a possible mechanism described in Figure 4b. As is depicted, during the reaction process, PLA-mPEG/Ca^2+ ^complex micelles first form, the calcium phosphate precipitation reaction takes place on the PLA-mPEG micelles, and then the resulting ACP nanoparticles aggregate on the polymer micelle skeletons to form hybrid nanoparticles. The polymer segments can act as the template and have a steric effect, the rapid growth of ACP can be inhibited, and the sizes of the ACP precipitate are confined to the nanometer scale range. During microwave-solvothermal treatment of ACP/PLA-mPEG hybrid nanoparticles in DMF, the dissolution of the polymer PLA-mPEG occurs and the nanopores form in ACP, resulting in the formation of ACP porous nanospheres.

**Figure 5:**
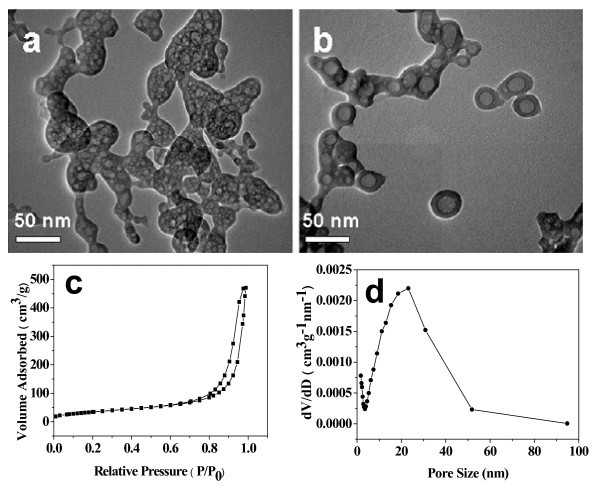
**Characterization of the as-prepared Eu**^**3+**^**:ACP (5 mol% Eu**^**3+**^**) porous nanospheres**: **(a, b) **TEM micrographs; **(c) **N_2 _adsorption-desorption isotherm; and **(d) **BJH pore size distribution curve.

The morphology of the as-prepared europium-doped Eu^3+^:ACP porous nanospheres were investigated with TEM (Figure 5a, b) at the doping percentage of 5 mol% Eu^3+^. From Figure 5, one can see that the as-prepared product consists of porous nanospheres, similar to the undoped ACP porous nanospheres. In a few cases, the nanosphere with a large pore in the center was also observed, as shown in Figure 5b. These results support the proposed formation mechanism shown in Figure 4b. Figure 5c, d exhibits the N_2 _adsorption-desorption isotherm and the corresponding Barrett-Joyner-Halenda (BJH) pore size distribution curve of Eu^3+^:ACP porous nanospheres. According to the International Union of Pure and Applied Chemistry, it can be classified as a type-IV isotherm loop which is the characteristic of the porous structure [47]. The BET-specific surface area (*S*_BET_), the BJH desorption cumulative pore volume (*V*_P_), and the average pore size are 126.7 m^2^/g, 0.53 cm^3^/g, and 16.7 nm, respectively.

**Figure 6:**
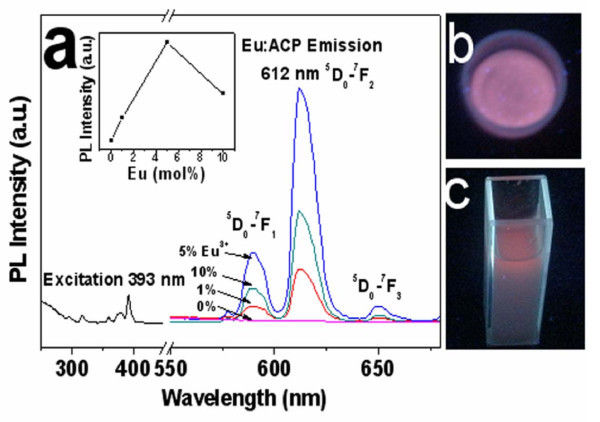
**PL properties of Eu**^**3+**^**:ACP porous nanospheres**: **(a) **Room-temperature excitation and PL emission spectra of Eu^3+^:ACP porous nanospheres; **(b) **the PL of the powder of Eu^3+^:ACP (5 mol% Eu^3+^) porous nanospheres under UV irradiation at 365 nm; and **(c) **the PL of Eu^3+^:ACP (5 mol% Eu^3+^) porous nanospheres dispersed in deionized water under UV irradiation at 365 nm.

PL excitation and emission spectra of Eu^3+^:ACP (5 mol% Eu^3+^) porous nanospheres measured at room temperature are shown in Figure 6a. To investigate the effect of the dopant content on PL, the concentration of Eu^3+ ^dopant varied in the range of 1 to 10 mol%. As shown in Figure 6a, the concentration of Eu^3+ ^dopant had almost no effect on the wavelength of emission peaks, but the PL intensity changed considerably by varying Eu^3+ ^concentrations. Three emission peaks appeared at about 590, 612, and 650 nm at an excitation of 393 nm. The most intense peak at 612 nm corresponds to the ^5^D_0 _→ ^7^F_2 _transition within Eu^3+ ^ions, while other peaks at 590 and 650 nm correspond to ^5^D_0 _→ ^7^F_1 _and ^5^D_0 _→ ^7^F_3 _transitions, respectively. The inset of Figure 6a displays the PL emission intensity at 612 nm as a function of the Eu^3+ ^concentration, and the highest PL emission intensity was observed at 5 mol% Eu^3+ ^doping. Under irradiation by UV lamp (365 nm), both powders of Eu^3+^:ACP (5 mol% Eu^3+^) porous nanospheres and dispersed suspension in deionized water show strong orange PL, as shown in Figure 6b, c.

**Figure 7:**
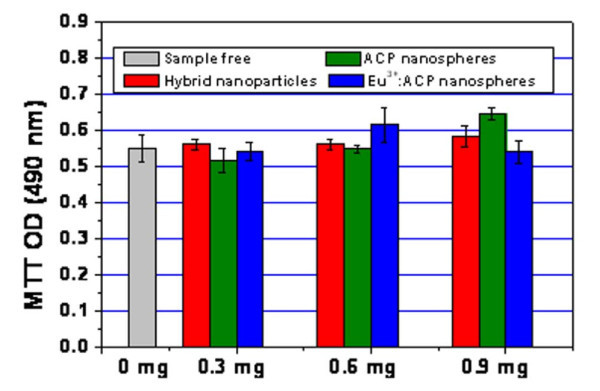
**Viabilities of PIECs which were cultured for 24 h with ACP/PLA-mPEG hybrid nanoparticles, ACP porous nanospheres, and 5 mol% Eu**^**3+**^**-doped ACP porous nanospheres at concentrations ranging from 0.3 to 0.9 mg/well**.

The nanostructures containing optically active rare earth ions have been of great interest in both fundamental studies and biomedical applications [1]. It is important to investigate the cytotoxicity of the rare earth-doped ACP porous nanospheres with endothelial cells, because these nanostructures have potential to be used in drug delivery and bio-imaging. The endothelial cells are a specialized type of epithelial cells which form the inner layer of blood vessels, and they are usually in contact with the biomaterials such as drug carriers. Therefore, the endothelial cells were chosen for the cytotoxicity study. Cell viability results (Figure 7) reveal that there were no cytotoxicity when the cells were subjected to ACP/PLA-mPEG hybrid nanoparticles, ACP porous nanospheres, and Eu^3+^-doped ACP porous nanospheres at concentrations ranging from 0.3 to 0.9 mg/well. The high biocompatibility can be explained by the chemical properties of these samples.

We investigated the drug loading and release behaviors of Eu^3+^:ACP porous nanospheres using a typical anti-inflammatory drug, ibuprofen. The drug loading capacity of Eu^3+^:ACP porous nanospheres was calculated from the absorbance values of ibuprofen hexane solution before and after drug loading. The drug loading capacity of Eu^3+^:ACP porous nanospheres reached 70 mg/g carrier.

**Figure 8:**
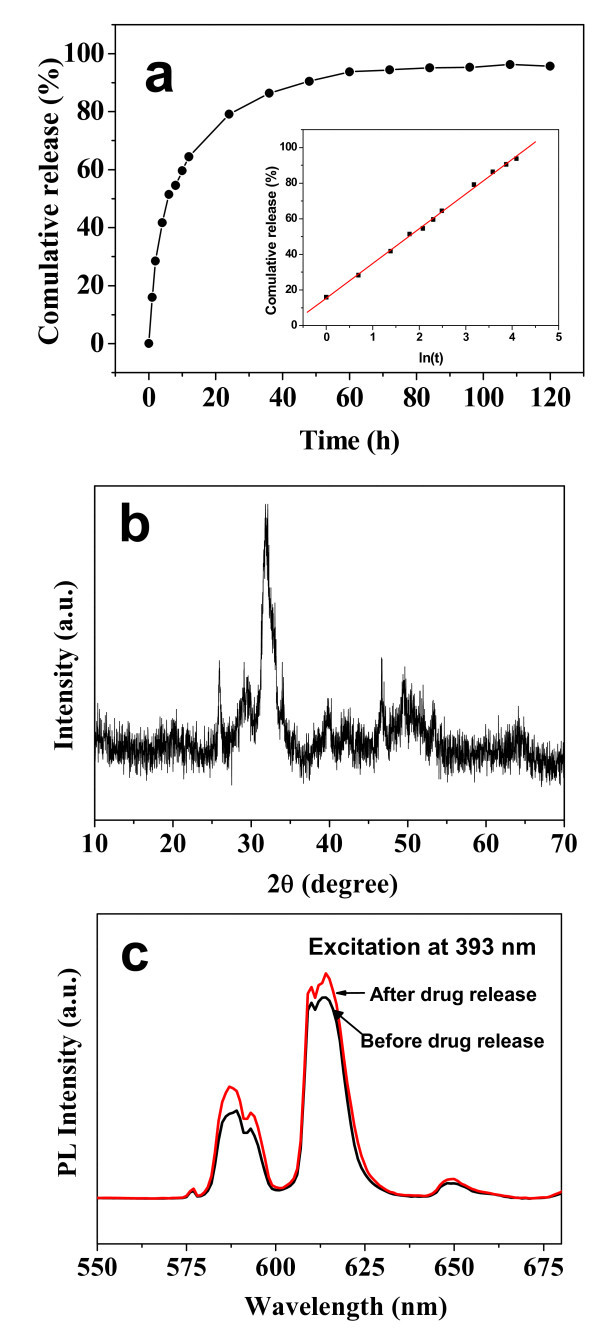
**Drug release and PL properties of ibuprofen-loaded Eu**^**3+**^**:ACP porous nanospheres**: **(a) **The drug release curve in SBF of a disk composed of ibuprofen-loaded Eu^3+^:ACP porous nanospheres, and the inset shows the cumulative drug release percentage versus natural logarithm of release time; **(b) **XRD pattern of the Eu^3+^:ACP porous nanosphere drug delivery system after drug release in SBF; and **(c) **room-temperature PL emission spectra of the drug loaded Eu^3+^:ACP porous nanospheres before and after drug release.

We also investigated the drug release behavior of Eu^3+^:ACP porous nanospheres in SBF. Figure 8a shows the drug release behavior of the disk composed of ibuprofen-loaded Eu^3+^:ACP porous nanospheres in SBF. The cumulative release amounts of ibuprofen from the disk were about 30, 40, 60, and 80% at the release times of 2, 4, 10, and 24 h, respectively. The final release amount reached about 96% at 120 h. In the sample preparation process, both dispersed Eu^3+^:ACP porous nanospheres and some agglomerated nanoparticles were formed. The experimental results of drug loading and release can be understood as due to the holistic behaviors of the sample consisting of dispersed Eu^3+^:ACP porous nanospheres and some agglomerated nanoparticles. One can see that the ibuprofen-loaded porous Eu^3+^:ACP nanosphere disk showed a slow and sustained release of ibuprofen, which could avoid the explosive release of ibuprofen and prolong the drug effect.

The inset of Figure 8a shows a relationship between the cumulative amount of released drug and the release time for the Eu^3+^:ACP porous nanosphere drug delivery system. The cumulative amount of released drug (*C*) has a linear relationship with the natural logarithm of release time (*ln*(*t*)), instead of the square root of time. A formula can be inferred for the drug release process of Eu^3+^:ACP porous nanospheres in SBF as follows:

(1) C=A+k ln(t)

This result is similar to that of our previous report on the drug release system of porous microspheres of calcium silicate hydrate [48]. For the drug delivery system of Eu^3+^:ACP porous nanospheres, the constant values of *A *and *k *are determined to be 15.47 and 19.46, respectively, with a regression factor of 0.999. Figure 8b shows the XRD pattern of the drug delivery system of Eu^3+^:ACP porous nanospheres after drug release in SBF, from which one can see that the product after drug release was HAp instead of ACP, indicating that the phase transformation from ACP to HAp occurred during the drug release process in SBF.

It is well acknowledged that the Higuchi model (*C *= *K*·*t*^1/2^) can well describe the kinetics of drug release from carrier materials [49-51], with a linear relationship between the cumulative amount of the released drug (*C*) and the square root of time (*t*^1/2^), and that the drug release is governed by a diffusion process. However, for the drug delivery system of Eu^3+^:ACP porous nanospheres, Eu^3+^:ACP porous nanospheres are bioactive, and they can transform from ACP to HAp in SBF solution during the drug release process. Therefore, for the drug delivery system of Eu^3+^:ACP porous nanospheres in SBF, the drug release kinetics is different from that of the model for common carrier materials.

In a previous study [28], the authors prepared HAp nanostructured porous hollow ellipsoidal capsules which were constructed by nanoplate networks using inorganic CaCO_3 _template. CaCO_3 _ellipsoids were synthesized by the reaction between Ca(CH_3_COO)_2 _and NaHCO_3 _in mixed solvents of water and ethylene glycol at room temperature, and they were used as the Ca^2+ ^source and cores. Then, the PO_4_^3- ^source was added to react with CaCO_3 _to form a HAp shell on the surface of CaCO_3 _ellipsoids. Dilute acetic acid was used to remove the remaining CaCO_3 _cores. The drug loading and release behaviors of HAp hollow ellipsoidal capsules were also investigated. The ibuprofen-loaded HAp hollow ellipsoidal capsules showed a slow and sustained release of ibuprofen in SBF. A linear relationship between the cumulative amount of the released ibuprofen and the square root of time was found for the first 12 h. Compared with HAp nanostructured hollow ellipsoidal capsules, the Eu^3+^:ACP porous nanosphere drug delivery system also showed a slow and sustained drug release profile. However, the IBU/Eu^3+^:ACP porous nanosphere drug delivery system exhibited a faster drug release rate than that of HAp nanostructured hollow ellipsoidal capsules. On the other hand, the drug release kinetics is different for the two drug delivery systems. For the IBU/Eu^3+^:ACP porous nanosphere drug delivery system, the cumulative amount of the released drug has a linear relationship with the natural logarithm of release time (*ln*(*t*)), instead of the square root of time. However, for IBU/HAp nanostructured hollow ellipsoidal capsule drug delivery system, a linear relationship between the cumulative amount of the released ibuprofen and the square root of time was found. The different drug release properties for the two drug delivery systems may be explained by the different chemical compositions, structures, and morphologies of the two samples.

Figure 8c shows PL emission spectra of drug-loaded Eu^3+^:ACP porous nanospheres before and after drug release. The characteristic emission peaks are still obvious in the emission spectra for both the samples. There were essentially no shifts in the spectral positions for the characteristic emission peaks, but the PL peak relative intensity and the shape of peaks changed after drug loading. The change of PL properties may be explained by the effect of ibuprofen. However, after *in vitro *drug release, it is worth noting that the intensities of the emission peaks increased. This phenomenon may be explained by the transformation of carrier material from ACP to HAp during the process of drug release in SBF.

The as-prepared Eu^3+^:ACP porous nanospheres have a porous structure, which is favorable for a sustained drug release. The Eu^3+^:ACP porous nanospheres empowered by luminescent properties show a great potential for applications in both drug delivery and in vivo bioimaging. Considering the biocompatible and biodegradable nature, Eu^3+^:ACP porous nanospheres are a new kind of promising biomaterial.

## Conclusions

Calcium phosphate is the most important inorganic constituent of biological hard tissues. ACP, as one of the most important calcium phosphates, is bioactive with biocompatibility and biodegradability. In this study, the authors report a facile method for the preparation of ACP/PLA-mPEG hybrid nanoparticles, which were successfully used as the precursor for the preparation of ACP porous nanospheres. PL function of ACP porous nanospheres was achieved by europium doping. The BET-specific surface area, the BJH-desorption cumulative pore volume, and the average pore size were 126.7 m^2^/g, 0.53 cm^3^/g, and 16.7 nm, respectively, for the Eu^3+^:ACP porous nanospheres. The experimental results of PL, cytotoxicity, as well as *in vitro *drug loading and release showed that the as-prepared Eu^3+^:ACP porous nanospheres were biocompatible and bioactive with favorable properties of PL, drug loading and drug release, implying that Eu^3+^:ACP porous nanospheres are a new kind of promising biomaterial with bi-functions of both luminescence and drug delivery.

## Abbreviations

ACP: amorphous calcium phosphate; BET: Brunauer-Emmett-Teller; BJH: Barrett-Joyner-Halenda; DMF: *N*, *N*-dimethylformamide; HAp: hydroxyapatite; PIECs: porcine iliac artery endothelial cells; PL: photoluminescence; PLA-mPEG: polylactide-block-monomethoxy(polyethyleneglycol); SBF: simulated body fluid.

## Competing interests

The authors declare that they have no competing interests.

## Authors' contributions

All listed authors contributed to the work of this article.
